# The relationship between sex change and reproductive success in a protandric marine gastropod

**DOI:** 10.1038/srep29439

**Published:** 2016-07-07

**Authors:** Antonio Brante, Adriana Quiñones, Francisco Silva

**Affiliations:** 1 Departamento de Ecología, Facultad de Ciencias, Universidad Católica de la Santísima Concepción, Chile; 2 Centro de Investigación en Biodiversidad y Ambientes Sustentables (CIBAS), Universidad Católica de la Santísima Concepción. Casilla 297, Concepción, Chile

## Abstract

Protandric species switch sex during their lifetime. According to theory, the time (body size) at which sex change occurs is determined by the reproductive success of individuals affected by social interactions as well as by post-copulatory factors. Experimental evidence is biased to few social systems making the exploration of general patterns difficult. We used the protandric marine gastropod *Crepidula coquimbensis* that partakes in intrabrood sibling cannibalism to test the following hypotheses: 1. Male-male competition for access to females and sibling cannibalism determine male reproductive success; 2. Males with greater access to females and with higher reproductive success will have reduced growth rates and will delay sex change. Artificial aggregations with different social structures were constructed and male reproductive success was estimated by paternity analysis. The results supported our expectations showing that male competitive ability for access to the female, time spent by males in the copulatory position, and sibling cannibalism affect reproductive success and influence time to sex change, with less successful males hastening sex change. Also, males that spent more time in the copulatory position had reduced growth rates. Comparing these results with those reported for other sequential hermaphrodites provides evidence supporting general patterns of sex change in nature.

The wide diversity of reproductive strategies observed in nature has prompted the study of factors driving the evolution of this diversity. Sequential hermaphroditism (sex change over time) has been observed in many marine taxa from various phylogenetic origins^1^. According to the size-advantage hypothesis, sex change is favored by natural selection where reproductive success increases more quickly with size (or age) for one sex compared to the other^2^,^3^. It is predicted that sex change occurs at the size (or age) where the reproductive success curves of males and females intersect^3^,^4^. Thus, evaluating the factors that affect reproductive success is of primary importance in understanding the evolution of sex change.

Experimental studies of the evolution and diversity of sequential hermaphroditism have been biased towards protogynous fishes (sex change from female to male)^5^,^6^,^7^ and to a lesser extent to protrandrous shrimps (sex change from male to female)^8^,^9^. Studies of these groups suggest that the strength of social interactions drive sex changes with individuals delaying or accelerating sex change in order to increase reproductive success^10^,^11^,^12^,^13^. In contrast, evidence from the literature indicates that sex changes in protandrous mollusks are less complex^14^. For example, the body size of three protandric gastropod species has been shown to be the same at the time of sex change regardless of differences in the populations’ mean body size, density, and sex ratio^14^. In the same study, growth rate was found to have an important effect on the timing of sex change where males with high initial growth rates change sex sooner and at smaller sizes than those with slower initial growth rates. The authors of this study^14^ argue that this apparent simplicity could be the product of three factors: (1) differences in the evolution of protogyny in fishes and protandry in mollusks, (2) differences in the complexity of behavior exhibited by vertebrates and invertebrates, and/or (3) biases due to the focus of research efforts on fishes and other groups. The same authors highlight that analyses at small scales may be useful for uncovering underlying variability in sex change at population scales. Despite the work mentioned, the lack of information on sequential hermaphrodism in other social systems prevents the explicit evaluation of different hypotheses concerning the evolution of this reproductive strategy. Consequently, it is difficult to determine the real diversity of behavioral responses that occur as a result of sex change. In this context, marine protandric mollusks provide a relevant study model for understanding the evolution of sequential hermaphroditism in nature.

Studies of the factors affecting sequential hermaphroditism in general, and protandry in particular, must include the study of mating systems^15^,^16^,^17^. Copulatory behavior, male-male competition, sperm storage, and sperm competition determine the present and future reproductive output of polyandrous species, and thus these factors shape the reproductive curve^18^. One of the greatest limitations to studying the link between mating behavior and sex change is the lack of reliable estimators of effective individual reproductive success; this is especially the case for species with complex mating behaviors. Female reproductive success, in terms of the number of eggs/offspring produced, is in general easy to quantify. In contrast, the quantification of male reproductive success presents a methodological challenge for researchers given the difficulty of estimating male fertilization success. Usually, male reproductive success is quantified based on observations of copulation success. Subsequently, post-copulatory events such as sperm competition or sibling competition are often ignored^17^,^19^,^20^,^21^. In protogynous fish species, sperm competition may significantly bias the expected results of classical models of sequential hermaphroditism^6^,^7^. Fortunately, the development of molecular techniques has facilitated the study of reproduction; in particular, paternity analysis using microsatellite loci has been a useful tool to test different hypotheses in sex-changing species.

*Crepidula coquimbensis* (Calyptraeidae; Fig. 1) is a protandric marine gastropod with internal fertilization. Females encapsulate, lay, and brood 10–32 ovicapsules containing 45–121 eggs^22^. These ovicapsules are brooded for approximately 40 days between the female’s neck and foot^22^. *C. coquimbensis* is a direct developer which means that juveniles hatch from the capsules at the end of the incubation time. This species is gregarious; a male can often be seen on the right side of the shell of the female close to the genital papilla in the copulatory position. Usually, aggregations are formed by one female and two to six males cohabiting empty shells of other marine snails such as *Tegula atra* and *Argobuccinum sp.* As such, there is likely a strong bias in the sex ratio of the aggregations which is in agreement with theoretical models for protandric organisms^17^,^23^. Males that are not in the copulatory position are highly mobile, meanwhile females are sedentary and encapsulate and brood their eggs.

Studying two populations of C*. coquimbensis*, Brante and collaborators^24^ found that the timing of sex change may depend on local population conditions; body size at sex change was negatively correlated with the number of males in the aggregation and the aggregation sex ratio^24^. In another study, paternity analyses using microsatellite markers indicated that most of the males in the aggregation participated in the brood but with different percentages of contribution^25^. Also, the percentage of male participation did not differ among ovicapsules within a brood suggesting that females of *C. coquimbensis* mix the sperm before egg fertilization^25^.

Embryos of *C. coquimbensis* have been shown to cannibalize siblings as intracapsular development progresses^22^,^26^. Cannibal embryos engulf, store, and digest capsule mates during embryonic development^22^,^26^. Approximately 10% of individuals survive the cannibalism and hatch as juveniles at the end of the ovicapsular period^22^. According to previous molecular studies, sibling cannibalism is not a random behavior in this species, and siblings from specific crosses show higher cannibalistic success than other combinations^26^. The high occurrence of multiple paternity in this species and the non-random cannibalistic behavior of embryos suggest that male reproductive success is not necessarily correlated with copulatory success or sperm competition success. In light of this evidence, we hypothesize for *C. coquimbensis* that: 1. Male-male competition for access to females (pre-copulatory factor) and intracapsular sibling cannibalism (post-copulatory factor) determines male reproductive success; 2. Males with greater access to the females for copulation (time in the copulatory position) and with higher reproductive success will have reduced growth rates and will delay sex change.

## Results

To test the different hypotheses proposed, experimental aggregations of *C. coquimbensis* with different social compositions were followed in the laboratory: i) 1 Female (F)+1 Male (M), ii) 1F+2M, iii) 1F+4M, iv) 1M and v) 2M. Male growth rate, time spent by different males on the female in the copulatory position, male reproductive success, and the time to, and body size at, sex change were recorded for two consecutive ovicapsule laying events and until the first male sex change occurred. Male reproductive success was estimated by conducting paternity analyses on embryos from two consecutive ovicapsule laying events. The main statistics and the probability of exclusion for the five microsatellite loci used for the paternity analysis are reported in Table 1. All females were virgins, since the results of the 1F+1M treatment indicated that the available male fathered all embryos in the brood. Paternity analyses performed on treatments 1F+2M and 1F+4M corroborated multiple paternity in this species.

### The effect of pre- and post-copulatory factors on male reproductive success

To test the hypothesis that male-male competition for access to females and intracapsular sibling cannibalism determine male reproductive success in *C. coquimbensis*, three lines criteria were examined: first, for all of the experimental aggregations where a female was present (1F+1M, 1F+2M and 1F+4M), we compared the number of males observed in the copulatory position and the number of males participating in paternity at pre- and post-cannibalism stages; second, we compared the reproductive success of males in terms of % of contribution to the brood, before and after cannibalism. Third, we evaluated if access to females in terms of time that the males spent in the copulatory position is a good predictor of male reproductive success.

Records on the behavior of males showed that at least one male was observed in the copulatory position during the experiment. For all of the 1F+1M treatment replicates, the male assumed the copulatory position with the female. Meanwhile, in 60% of the replicates for the 1F+2M treatment, both males independently assumed the copulatory position with the female. In the 1F+4M treatment, two males independently assumed the position with the female in 80% of replicates, and in the remaining cases, three males were observed in the copulatory position during the entire experimental period. The time spent by a male on the female was highly variable within and between treatments: the maximum time spent on the female was 276 days in the 1F+1M treatment, while the mean time spent was 75.1 (±84.12 SD), 136.4 (±86.16 SD), and 267 (±70.01 SD) days for the 1F+4M, 1F+2M, and 1F+1M treatments, respectively.

Results of the 1F+2M treatment indicated there was no significant difference in the number of participant males between the different stages (copulatory position, and pre- and post-cannibalism) in the first laying event nor in the second event (P > 0.027 in all cases; Fig. 2A); however, there was a difference between the number of males observed in the copulatory position and the number of males participating as fathers in the pre-cannibalism stage for the first laying event (paired-samples t-Test: t = −3.67, df = 9, P < 0.027; Fig. 2A). For the 1F+4M treatment during the first laying event, on average, there was evidence for an increase in the number of males recorded in the copulatory position and the number of males fathering embryos at the pre-cannibalism stage (paired-samples t-Test: t = −3.20, df = 9, P < 0.025; Fig. 2B). However, significantly fewer males were detected as fathers after cannibalism than were detected pre-cannibalism (paired-samples t-Test: t = 2.68, df = 9, P < 0.025; Fig. 2B). A similar pattern was observed for the second laying event (in both cases P < 0.025; Fig. 2B).

As we expected, intracapsular sibling cannibalism not only may affect the number of males participating in the brood but also the relative contribution of different males. The different percentages of contribution of males at pre- and post-cannibalism stages in treatments 1F+2M and 1F+4M oscillated between positive and negative values (Fig. 3). This means that some males were overrepresented (positive values) in the brood after cannibalism, with offspring from specific parental crosses having higher cannibalistic abilities than others. These results suggest that intracapsular sibling cannibalism affects the reproductive success of *C. coquimbensis* males in different ways.

The General Lineal Model run on the percentage of male participation (considering both laying events) with social composition as a fixed factor, and time spent by the males in the copulatory position as a covariable, explained 64% of the observed variance. Results showed that only the time in the copulatory position was significant (F_1,24_ = 30.8, P < 0.001). In accordance with our expectations, the positive coefficient of the time in the copulatory position covariable (0.44) indicated that *C. coquimbensis* males with higher competitive abilities to monopolize the female in copulation have higher reproductive success than other less competitive males in the aggregation, and this is irrespective of the social composition structure (Fig. 4).

### Male reproductive success, growth rate and sex change strategy

To test the hypothesis that males with greater access to females for copulation and with higher reproductive success will have reduced growth rates and will delay sex change, we measured the relationship between growth rate and time in the copulatory position, male reproductive success, and time to sex change in the different social composition treatments. The results showed that the growth rate of the first male to change sex in the aggregation was highly variable within and between treatments ranging from 0.002 to 0.044 mm/day for the five treatments, with the highest rates observed in the 1M treatment (Fig. 5A).

The General Lineal Model run to evaluate the effect of time in the copulatory position on male growth rate for the different social composition treatments where a female was present, explained 68% of the observed variance. Social composition as a fixed factor, time in the copulatory position as a covariable, and the interaction between both of these, were significant (GLM: Social composition (SC): F_2,64_ = 5.91, P < 0.01; Time in the copulatory position (TCP): F_1,64_ = P < 0.001; SC × TCP: F_2,64_ = P < 0.001). The lowest growth rates were observed in the 1F+1M treatment where no male-male competition existed, and where the single male present in the aggregation passed most of its time on the female in the copulatory position (Fig. 5B). As a general pattern and as we hypothesized, the negative coefficient of the time in the copulatory position covariable (−0.0001) indicates that there is an inverse relationship between male growth rate and the time spent by the male on the female in the copulatory position; males grew slower when they were in the copulatory position.

The time that it took for a sex change to occur varied significantly within and between treatments. As we expected, the fastest sex changes were observed in the two treatments where females were absent (1M and 2M; male reproductive success = 0), with means of 55.04 and 53.6 days, respectively. Meanwhile, the slowest sex change, 93.3 days on average, occurred in the 1F+2M treatment (One-way ANOVA: F_4,45_ = 97.83, P < 0.01; Tukey *a posteriori* test P < 0.05; Fig. 6A). Interestingly, there was a narrow range of shell sizes at which sex change occurred; body size was between 6.24 and 9.33 mm on average. Furthermore, the largest significant body size at sex change was observed in the 1F + 1M treatment (F_4,45_ = 4.50, P < 0.01; Tukey *a posteriori* test P < 0.05; Fig. 6B). As we hypothesized, the Fisher’s exact tests show that there is a significant relationship between the ranking of time to change sex and the ranking of male reproductive success; males with lower reproductive success changed sex sooner than successful males (Fisher’s exact test: 1F+2M: Pre-cannibalism: χ^2^ = 5, df = 1, P < 0.05; Post-cannibalism: χ^2^ = 10.21, df = 1, P < 0.05. 1F + 4M: Pre-cannibalism: χ^2^ = 24.74, df = 3, P < 0.05; Post-cannibalism: χ^2^ = 22.25, df = 3, P < 0.059; Fig. 7).

## Discussion

This is the first work providing direct evidence of the relationship between social interactions, male reproductive success, and the timing of sex change for a protandric gastropod. In *C. coquimbensis*, the timing of and the body size at which sex change occurs were found to be plastic and varied according to the social interactions that the individual experienced in the aggregation. More specifically, results supported our hypotheses that reproductive success and the timing of sex change is determined by male competitive ability for access to females for copulation and intracapsular sibling cannibalism. As a general pattern, we found that males that spent more time in the copulatory position had higher reproductive success, lower growth rates, and delayed sex change in comparison with less successful males which changed sex sooner.

The variance and invariance of body size at sex change has been largely discussed in the literature^16^,^27^,^28^,^29^,^30^,^31^,^32^. Within the calyptraeid group, body size at sex change observed at intra- and inter-specific levels is highly variable^30^. According to Collin^33^, such variability may be explained by differences in the characteristics of the social aggregations that individuals experience during their lifetime. This, in turn, may affect male reproductive success and the optimal size for a sex change. Here we found that the body size of the first male that changed sex in the aggregation varied according to the social configuration of the aggregation. Moreover, when the remaining males of the group were considered, the body size at sex change varied even more; sex change occurred in individuals that were between 6.24 and 12.4 mm in size, which corresponds to 20–41% of the maximum body size observed for this species (30 mm approximately in this study and in Véliz *et al.*^22^). The largest sizes at time of sex change were recorded in males of treatment 1F+1M where no male competition for the female occurred, and a single male monopolized the female in the copulatory position. In this way, the results presented here suggest that when a female is not present or is more or less monopolized by another male (high levels of male-male competition), *C. coquimbensis* will change sex at smaller body sizes than when competition is low.

Male growth rate varied greatly, between 0.0006 and 0.01 mm/day. As we expected, growth rate was negatively affected by the time that the male spent on the female in the copulatory position. Other studies of calyptraeidae species have shown that male growth rate and time to sex change is influenced by the presence of a female in the aggregation. For example, *Crepidula fornicata* males kept in isolation from females change sex at smaller sizes than males in direct contact with a female^34^. Similarly, *Crepidula* cf. *marginalis* males in physical contact with a female have reduced growth rates and delayed sex change^34^. Inhibition by the female could also explain the longer time to sex change observed for *C. coquimbensis* males in the 1F+1M treatment given the monopolization of the female by the only male present in the aggregation. Physical stimulation and chemical inhibition have been suggested by Carrillo-Baltodano & Collin^35^ for *C.* cf. *marginalis*. Alternatively, competition for food could influence growth rate given that this species is a filter feeder and feeding could be affected by the proximity of the female and the male in the copulatory position. Likewise, male feeding might be impeded by copulation. Alternatively, males that have less access to the female may allocate more energy to growth in order to outcompete other males in the aggregation. Specific and complex experimental designs are required to test these hypotheses.

Theory suggests that reproductive success shapes the optimal timing of sex change. Our results for *C. coquimbensis* reveled an inverse relationship between the ranking of sex change and the ranking of male participation in the brood. For protandric marine gastropods, there are few studies that use realistic estimates of reproductive success (paternity analyses) to evaluate sex allocation theory hypotheses. For one of the few studies that does exist, combined observational and molecular data for *C. fornicata* indicates that male reproductive success increases with body size^18^. If bigger males delay sex change, *C. fornicata* males with higher reproductive success should change sex later than unsuccessful males. Thus, the reproduction of *C. fornicata* and *C. coquimbensis* (this study) is in concordance with the expectations of sex allocation theory: low-ranking males change sex because their expected reproductive return is low. Nevertheless, the aggregations of these two species differ in terms of their social structure. Specifically, it is common to observe more than two females and many males in *C. fornicata* aggregations while more than 85% of host shells in *C. coquimbensis* aggregations are occupied by only one female and two to four males^24^. From these observations, a conundrum arises considering the evolution of reproduction in *C. coquimbensis*. If one male often monopolizes the female, why aren’t there more females in the aggregation as a product of the remaining unsuccessful males that change sex earlier? As stated above, *C. coquimbensis* aggregations inhabit empty shells of other gastropods cohabiting with *Pagurus* hermit crabs. Previous studies have shown that *C. coquimbensis* males may colonize different host shells, often taking advantage of the gregarious behavior of hermit crabs during mating or feeding periods^36^. Given the space limitations of the host shells, it is possible that less successful *C. coquimbensis* males leave the host and then change sex if the new host is unoccupied; alternatively, these individuals may remain male if the new host already houses a female. Additional experimental work is needed to test this hypothesis.

The strategy of changing sex is common in many marine species and although different evolutionary histories exist, general patterns may be observed. In this study of *C. coquimbensis,* we demonstrate that, as theory predicts, the timing of and body size at which sex change occurs are plastic traits that are strongly associated with male reproductive success. Specifically, male-male competition, male-female interactions, sperm storage by females, and embryo cannibalism drive timing of sex change in this species.

## Methods

### Sample collection

Individuals of *Crepidula coquimbensis* were collected from Puerto Aldea (30°17′32″S, 71°36′30″W) located on the north-central coast of Chile. Specimens were transported in coolers to the laboratory of the Faculty of Science of the Universidad Católica de la Santísima Concepción.

### Laboratory experiments

**Figure 1:**
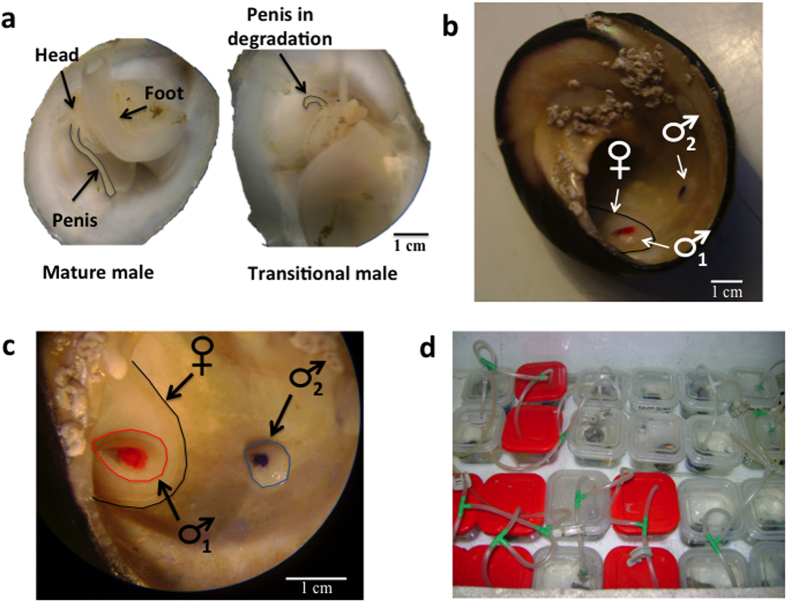
(**a**) Photographs of *C. coquimbensis* showing anatomical details of individuals at the male stage and transitional stage (changing from male to female). (**b**) One female and two males (individuals with red and blue spots) inside of a host shell of the marine gastropod *Tegula sp*. (**c**) A close up of the individuals inside the host shell. (**d**) Plastic boxes used to maintain the experimental aggregations of *C. coquimbensis*.

**Figure 2:**
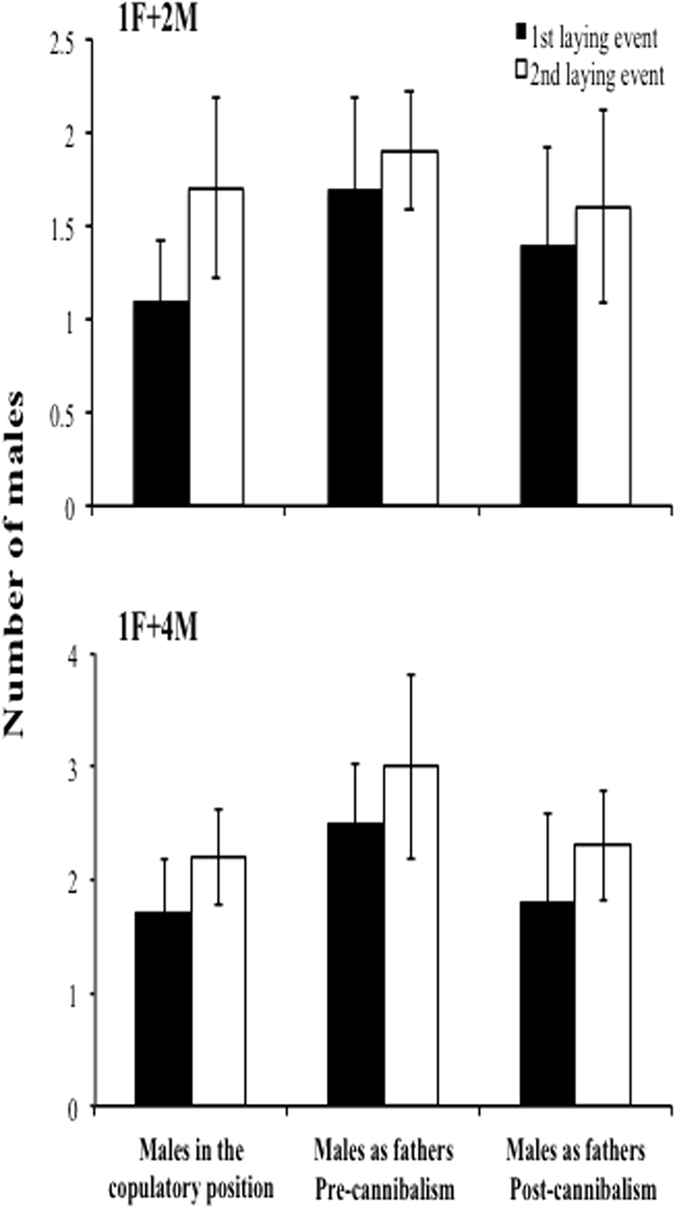
Number of *C. coquimbensis* males observed in the copulatory position in the treatments 1F+2M, 1F+4M. Also, the number of males fathering the brood, estimated from paternity analysis, is presented. Estimates include two ovicapsule laying events and before and after embryonic cannibalism. Vertical lines on each bar correspond to 1 standard deviation.

**Figure 3:**
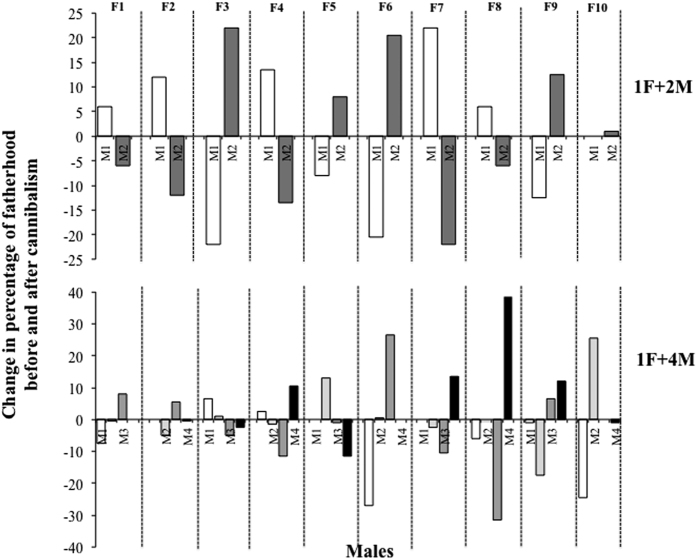
Change in the reproductive success of *C. coquimbensis* males during intracapsular development as a product of embryo cannibalism. Positive values represent an increase in participation in fathering the brood; negative values represent a decrease in participation in fathering the brood.

**Figure 4:**
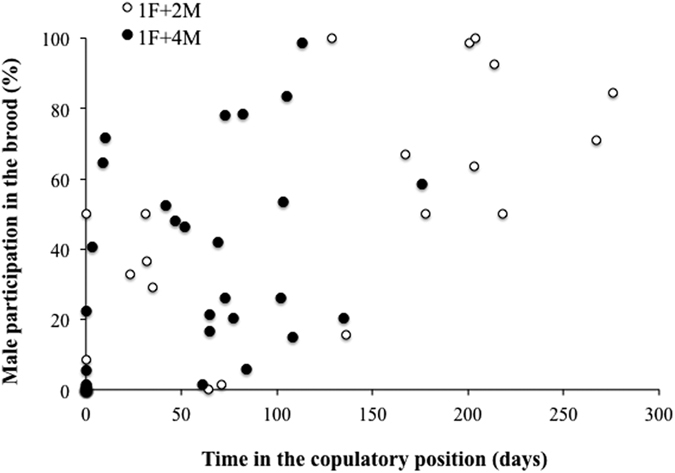
The relationship between the time spent by males in the copulatory position and male reproductive success in terms of its relative contribution to the brood measured after embryo cannibalism for *C. coquimbensis*. Results for treatments 1F+2M and 1F+4M are shown.

**Figure 5:**
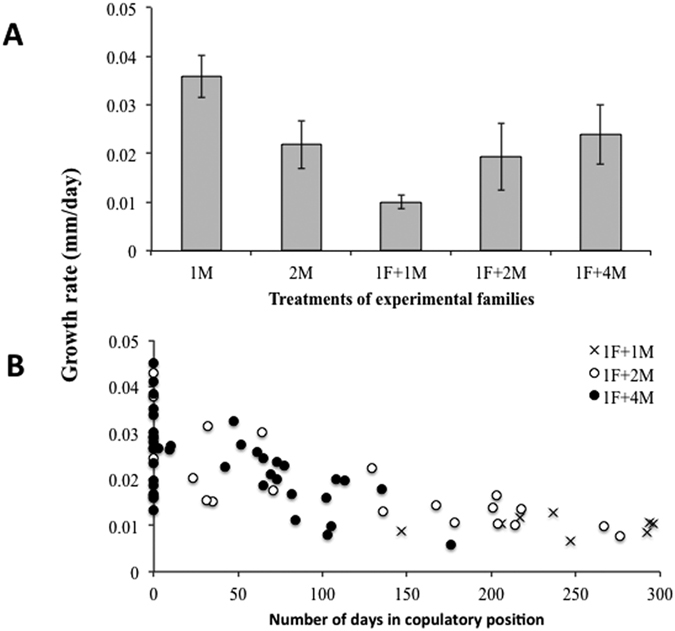
(**A**) Growth rate (mm/day) of the first *C. coquimbensis* male to change sex observed in the different experimental treatments. Vertical lines on each bar correspond to 1 standard error. (**B**) Relationship between growth rate and time in days that the males spent on the female in the copulatory position in three social composition treatments where a female was present.

**Figure 6:**
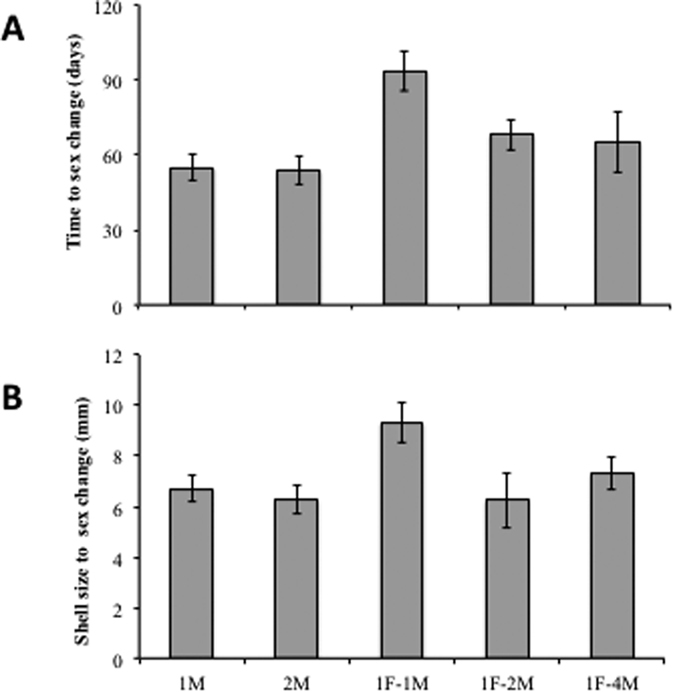
The average time (**A**) and body size (**B**) at which sex change occurred for *C. coquimbensis* males placed in differing social aggregations. Vertical lines on each bar correspond to 1 standard deviation.

**Figure 7:**
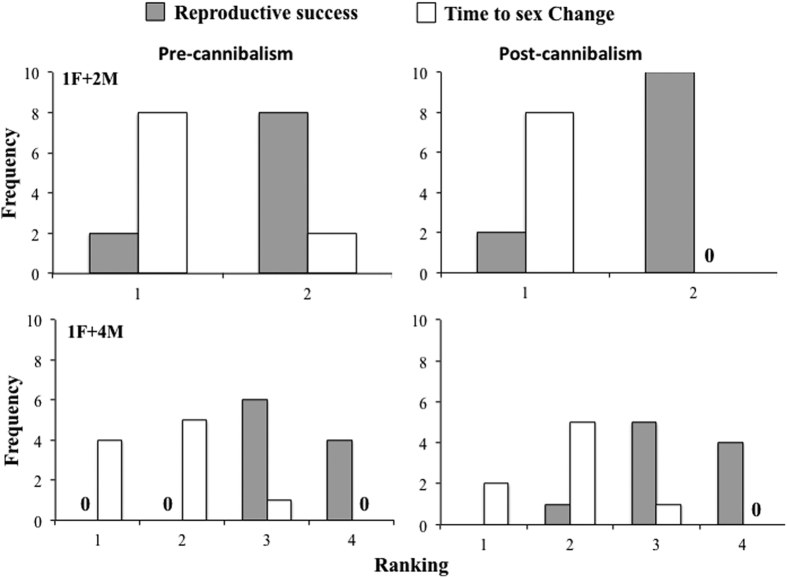
Frequency of male individuals observed at different rankings of reproductive success and time to sex change in two experimental social aggregations, 1F+2M and 1F+4M, of *C. coquimbensis*. Results are shown for estimations before and after intracapsular cannibalism. A ranking of 1 for reproductive success corresponds to the male with the highest value, meanwhile, a ranking of 1 for sex change corresponds to males that changed earliest.

To obtain virgin males and females, in the laboratory, hatching juveniles of approximately 1 mm in size were separated individually in plastic boxes with constant aeration and temperature (14 °C). The juveniles were fed twice a day with aliquots of cultured phytoplankton *Isochrysis galbana*. The sexual stage of the individuals was recorded weekly; the individuals were sexed according to the presence of sexual organs^24^,^37^. Males and females were classified by the presence or absence of a penis and female genital papilla (Fig. 1a); transitional individuals (changing from male to female) had both a penis and female genital papilla or were >8 mm and lacked a penis or female genital papilla^24^ (Fig. 1b). Male maturity was reached after 4–5 weeks, and females were obtained after 1–2 months. None of the individuals bypassed the male stage. Minimum shell length recorded for males was 4 mm and for females was 8 mm; this is in concordance with body sizes recorded in the field by Brante *et al.*^24^ at the same site.

To evaluate different hypotheses proposed, experimental families (aggregations) with different social compositions were arranged in five treatments: i) 1 Female (F)+1 Male (M), ii) 1F+2M, iii) 1F+4M, iv) 1M and v) 2M. Only the first four treatments were used to evaluate the effect of male interactions on sex change. Treatments iv) and v) were used to evaluate the influence of female presence on sex change. The social compositions used in these experiments simulate the sex ratio observed in natural populations of this species^24^. To avoid body size as a confounding factor, we used individuals with a narrow range of shell lengths; male shells ranged from 5 to 6 mm and female shells ranged from 17 to 18 mm. All treatments were carried out with virgin males and females, and each male was marked with indelible ink of different colors for identification and recording behavior (Fig. 1b,c). To simulate the natural environment, each experimental family was located in an empty host shell of the marine gastropod *Tegula atra*, which were placed in plastic boxes filled with seawater at 15 °C (±1 °C) that were constantly aerated using air stones and air pumps (Fig. 1d). Individuals were fed twice a day with aliquots of cultured phytoplankton (*Isochrysis galbana*). The experimental families were followed for a maximum of approximately 320 days until two ovicapsule laying events were sampled or until the first male changed sex. Ten replicates per treatment were carried out.

### Pre- and post-copulatory factors, and reproductive success

The effect of pre- and post-copulatory factors on male reproductive success of *C. coquimbensis* was evaluated in treatments 1F+1M, 1F+2M and 1F+4M during the two reproductive events (capsule laying) studied. For this, the identity of males observed in the copulatory position (right side of the female) was recorded two times a day (in the morning and evening), and the total time (days) in the copulatory position was estimated. In addition, the number of males participating as fathers and the percentage of contribution of each father to the brood before and after cannibalism were estimated by paternity analysis (see below).

Ovicapsules from brooding females were collected for all female replicates at the beginning of the incubation period containing early stage embryos (before sibling cannibalism) and at the end of the embryonic development before hatching (after sibling cannibalism). As it has been shown that *C. coquimbensis* appears to mix sperm before fertilization, the parentage distribution of participating males is well represented inside each ovicapsule^24^. Given this and the time constraints of genotyping microsatellites, we intensified sampling inside capsules rather than between capsules to increase the probability to detect rare fathers. We followed the same sampling protocol previously used by Brante and collaborators^25^ for this species. Three ovicapsules per brood were chosen representing roughly 10 to 15% of the total number of ovicapsules per brood. The ovicapsules were haphazardly selected and removed from each female at both embryonic stages for the two ovicapsule laying events recorded. From each ovicapsule, between 30 and 50 embryos were preserved in ethanol (95%) for subsequent paternity analyses; this represented 40–65% of the total embryos in an ovicapsule. Finally, the cephalic portion of each adult was individually stored in alcohol (95%) for genetic analyses.

For paternity analyses, we pooled the embryos of the three capsules sampled, and we estimated the total number of fathers in the clutch and the percentage of representation of each father in the clutch as the number of embryos fathered by the corresponding male divided by the total number of embryos analyzed in the clutch. Both variables were estimated for the offspring before and after cannibalism and for the two laying events.

The number of males observed on the female in the copulatory position and the number of males participating in the broods before and after cannibalism were compared with paired-sample t-Tests performed for each experimental aggregation treatment and laying event. This is because the number of males recorded in the copulatory position and fathering embryos are not independent. Given that multiple comparison were run on the same data, the false discovery rate approach modified by Benjamini and Yekutieli^38^ was used to estimate the critical probability value. Statistical assumptions of homoscedasticity and normal distribution were met in the raw data. We also compared the contribution of males to the brood pre- and post-cannibalism calculating the differences in male participation as follow: % male contribution post-cannibalism-% male contribution pre-cannibalism. Positive values are interpreted as higher relative contribution of a male (reproductive success) in relation to other males as a product of an offspring with higher cannibalistic abilities^26^.

Finally, to evaluate if the time spent by the male on the female is a good predictor of the final reproductive success of the male (after sibling cannibalism), a General Lineal Model was run on the percentage of male participation (considering both laying events) with social composition treatments as a fixed factor (considering only treatments with more than one male present (1F+2M and 1F+4M)), and time spent by the males in the copulatory position as a covariable; the interaction term between the social composition factor and time in the copulatory position was also evaluated. Statistical assumptions of normal distribution and homoscedasticity were met in the raw data.

### Male reproductive success, growth rate and sex change strategy

To test if males with high reproductive success had reduced growth rates and delayed sex change, body size and sex were recorded weekly in all of the experimental aggregations essayed. Each individual was measured with a caliper to the nearest 0.01 cm and sexed as described above. In the case of treatments where a female was present, the time spent by males on the female was also recorded. Individuals in the copulatory position were assumed to be males to avoid stressing the individuals and affecting sex change patterns.

To evaluate the effect of the time in the copulatory position on male growth rate, a General Lineal Model was run with the social composition treatments (1F+1M, 1F+2M and 1F+4M) as the fixed factor and time spent by the males in the copulatory position as a covariable; the interaction term between social composition factor and time in the copulatory position was also evaluated. The raw data met statistical assumption of normality; however, the data were root square transformed to meet homoscedasticity requirements.

The timing of sex change for the different social composition treatments was compared using a one-way ANOVA. Tukey *a-posteriori* tests were performed when significant effects were detected. The statistical assumptions of normality and homoscedasticity were met in the raw data. To determine if males with lower reproductive success change sex earlier, the relationship between the ranking of time to change sex and the ranking of male reproductive success was evaluated with Fisher’s exact tests using the frequency of both rankings; the 1F+2M and 1F+4M treatments and the data at pre- and post-cannibalism stages were considered separately.

### Paternity analysis

Paternity analyses were run using the same five microsatellite loci used in Brante *et al.*^25^,^26^. These loci are highly variable and have a high overall probability to exclude a randomly chosen unrelated candidate parent from parentage given the genotype of the offspring and of the mother (Probability of exclusion >0.95). Offspring paternity analysis was performed using the software CERVUS 2.0^39^ based on the procedure proposed by Meagher (1986)^40^ using maximum likelihood calculations. Given the genotypes of the embryos, of their known mothers (the brooding female), and of the potential fathers (males in the aggregation), paternity was assigned to the male with the highest log-likelihood ratio (LOD). One advantage of using CERVUS 2.0 is the possibility to assess the statistical significance of LOD through computer simulations^39^. These computations were carried out using 10,000 iterations based on population allelic frequencies estimated using the genotypes of the 100 adults studied.

## Additional Information

**How to cite this article**: Brante, A. *et al.* The relationship between sex change and reproductive success in a protandric marine gastropod. *Sci. Rep.* **6**, 29439; doi: 10.1038/srep29439 (2016).

## Figures and Tables

**Table 1:** Polymorphism of the five microsatellite loci for the 130 adults (males and females from all experimental aggregations) used for the paternity analysis.

Locus	N_a_	H_e_	H_o_	P_HW_	P_excl_
CcoqAC2F4	15	0.813	0.799	0.711	0.401
CcoqCT1D10	25	0.722	0.786	0.582	0.562
CcoqCT1F4	28	0.890	0.912	0.311	0.790
CcoqCT1H5	17	0.935	0.899	0.421	0.498
CcoqCT3F2	19	0.868	0.908	0.274	0.810
Over all loci	20.8	0.845	0.854	0.461	0.982
Standard deviation	4.9	0.081	0.072		

Locus name, number of observed alleles (N_a_), unbiased expected (H_e_) and observed (H_o_) heterozygosity are indicated for each locus and over the 5 loci used. The probability value of an exact test for the null hypothesis of Hardy-Weinberg equilibrium (P_HW_) and the exclusion probability (P_excl_, at 95% of confidence level) defined as the probability of excluding a randomly chosen unrelated candidate parent from parentage given the genotype of the offspring and of the mother are indicated for each locus and over all loci.
